# Design of a Multi-Sensor Cooperation Travel Environment Perception System for Autonomous Vehicle

**DOI:** 10.3390/s120912386

**Published:** 2012-09-12

**Authors:** Long Chen, Qingquan Li, Ming Li, Liang Zhang, Qingzhou Mao

**Affiliations:** 1 State Key Laboratory of Information Engineering in Surveying, Mapping and Remote Sensing, Wuhan University, No.129, Luoyu Road, Wuhan, 430079, China; E-Mails: lchen.whu@gmail.com (L.C.); zl200531610254@126.com (L.Z.); qzhmao@whu.edu.cn (Q.M.); 2 School of Electronic Information, Wuhan University, No.129, Luoyu Road, Wuhan, 430079, China

**Keywords:** autonomous vehicle, travel environment perception system, multi-sensor cooperation, road and lane detection, traffic sign detection

## Abstract

This paper describes the environment perception system designed for intelligent vehicle SmartV-II, which won the 2010 Future Challenge. This system utilizes the cooperation of multiple lasers and cameras to realize several necessary functions of autonomous navigation: road curb detection, lane detection and traffic sign recognition. Multiple single scan lasers are integrated to detect the road curb based on Z-variance method. Vision based lane detection is realized by two scans method combining with image model. Haar-like feature based method is applied for traffic sign detection and SURF matching method is used for sign classification. The results of experiments validate the effectiveness of the proposed algorithms and the whole system.

## 1. Introduction

**Figure 1.**
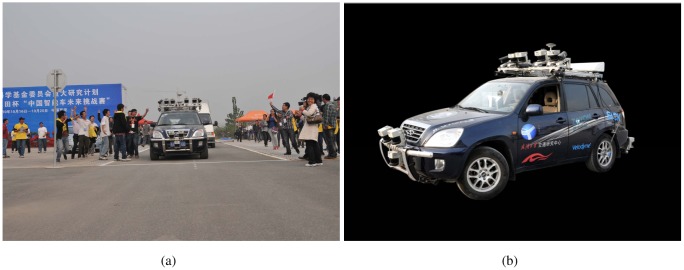
(**a**) At approximately 3:04 pm on Oct 18, 2010, SmartV-II was the first robot to complete the Future Challenge; (**b**) Autonomous Vehicle SmartV-II, developed by Wuhan University.

Intelligent Vehicle System (IVS) is a comprehensive system which should have several necessary functions: travel environment perception, self-localization, path planning and vehicle control. Travel environment perception is the foundation of other functions in IVS. This paper introduces the multi-sensor fusion travel environment perception system designed for our autonomous vehicle SmartV-II. The main functions include road curb detection, lane detection and traffic sign recognition. With the help of this travel environment perception system, SmartV-II became the only robot to complete comprehensive test section of the 2010 Future Challenge in time, see Figure 1.

IVS has been studied for a long time, especially since the DAPRA Challenge held in 2005. Many effective solutions have been proposed for road and lane detection and traffic sign recognition.

### 1.1. Road and Lane Detection

Road can be mainly divided into structured road and unstructured road based on the structure information. The former means regular road with visible lane markings, such as highway and most urban road. For structured road, lane detection and following as the key technology have been studied over last two decades. Some effective lane detection systems have been proposed, such as AWSTM, AutoVue, RALPH ([1–3]), AURORA [4], SCAR [5], GOLD ([6,7]), and LOIS [8]. These lane detection algorithms can be mainly grouped into two categories: edge based methods and model based methods. Edge based methods are most widely used [9,10].They are fast but highly dependent on the method used to extract the edges corresponding to the lane boundaries. When the road condition is complex, these methods may easily fail. Common road models include triangle model, straight line model, clothoid model, polynomial model and spline model, *etc.* Wang *et al.* [11] computed the likelihood probability through fitting the detected features to the model, and Kang *et al.* [12] and Wang *et al.* [13] found the extreme value of the energy function to the lane location, then the Kalman filter was used for predicting the parameters of the model. These algorithms would be time-consuming because of the iterative operation. Unstructured road refers the irregular road without normal markings such as campus and park road, rural road and off-road. In the situation, researchers mainly focus on the natural road boundary and drivable range detection [14–17]. Lieb *et al.* [14] used one-dimensional template matching and the sum of squared differences combined with optical flow to determine the most similar regions in front of vehicle. This method can hardly deal with the situation where there is an unexpected obstacle in the front. Dynamical sampling windows are used for training range detection in [15], but the selected range can not represent the real road classes feature space well. Our previous solution of lane detection is reported in [18]. In this paper, we apply a more believable method based on laser information for locating the road range, because the laser has more reliability depth information which is easier to find structural change. In [19], a trigonometry based road detection method using laser scanner is proposed, which applies the relationship of neighboring three laser points. However, because of the ranging error, the relationship may be destroyed and this method will be less robust as the range increases. In this paper, a Z-Variance based road curb detection method is proposed, which is range independent. Chen *et al.* [20] also introduced some recent developments of active vision in robotic systems.

### 1.2. Traffic Sign Detection and Recognition

Traffic sign detection and recognition in realtime is a vital issue in IVS and Driver Assistance System (DAS). One decade age, realtime performing systems have been successfully achieved [21–23]. Traffic sign recognition usually consists of two components: detection and classification. First, the location of the traffic signs are found and the target rectangles are extracted in the detection stage. To which category does the candidate sign belong is the main issue needing to be addressed in the classification phase. For traffic sign detection, color segmentation is the most common method. RGB color model is widely used [24]. RGB color space has a higher sensitivity to light intensity. Therefore, HIS and HSV which are not affected by the lighting changes have been used [25,26]. Some other authors also used YIQ [27], YUV, L*a*b [28] and CIE color spaces. Some authors developed databases of color pixels, look-up tables and hierarchical region growing techniques [26,29,30]. Shape based method is usually used for a final detection after the color segmentation. Many circle, ellipse and triangle detection methods also have been used. Soetedjo and Yamada [31] discussed ellipse detection in complex scene with neighborhood characteristics and symmetric features of the simple coding. Piccioli *et al.* [32] analyzed the color information and geometrical characteristic of the edges to extract possible triangular or circular signs. For traffic sign classification, many methods have been employed for traffic signs classification such as template matching, LDA, SVM, ANN and other machine learning methods. OCR systems are applied in [28,33,34] using the pictogram-based classification by template matching and cross-correlation. In [35,36], the authors make use of the LDA to distinguish between the road signs. The Multi-Layer Perception [37] is widely used in the current approaches. Neural networks are also widely adopted [38,39]. Support vector machines (SVM) are largely adopted to classify the inner part of road signs [40]. Random forests, an ensemble learning technique, are used in [41] to classify signs, and a comparison is made between this technique and SVM and AdaBoost. In recent years, one of the most accepted and widely used approach in object detection has been proposed by Viola and Jones [42]. Their approach is based on a cascade of detectors, where each detector is an ensemble of boosted classifiers based on the Haar-like features. Inspired by detector presented in [42], we apply this method combined with color segmentation for the traffic sign detection. Different from above solutions, this paper presents a low-cost multi-sensor integrated system to realize the necessary functions based on several novel algorithms. The contributions of this paper are as follows:

1. By reasonably arranging several simple low-cost sensors, our system can realize complex functions without high-end sensors. Combination of cameras and lasers based road detection method can deal with not only structured road but also unstructured road.
2. Multiple sensors are skillfully installed for covering more view around the vehicle to satisfy the situation that the vehicle drives with high speed or passes a turn with high curvature.
3. Traffic signs are divided into six classes; for each class, we trained a classifier based on Haar-like features for the detection and the scale invariant feature SURF is used for the sign classification.

The rest of the paper is organized as follows. Section 2 introduces the layout of the sensors. Section 3 describes Z-variance based road curb detection. Section 4 presents two scans method for multiple lanes detection. Realtime traffic sign recognition is introduced in Section 5. Experiments and results are discussed in Section 6. Conclusions are given in Section 7.

## 2. Multi-Sensor Layout

**Figure 2.**
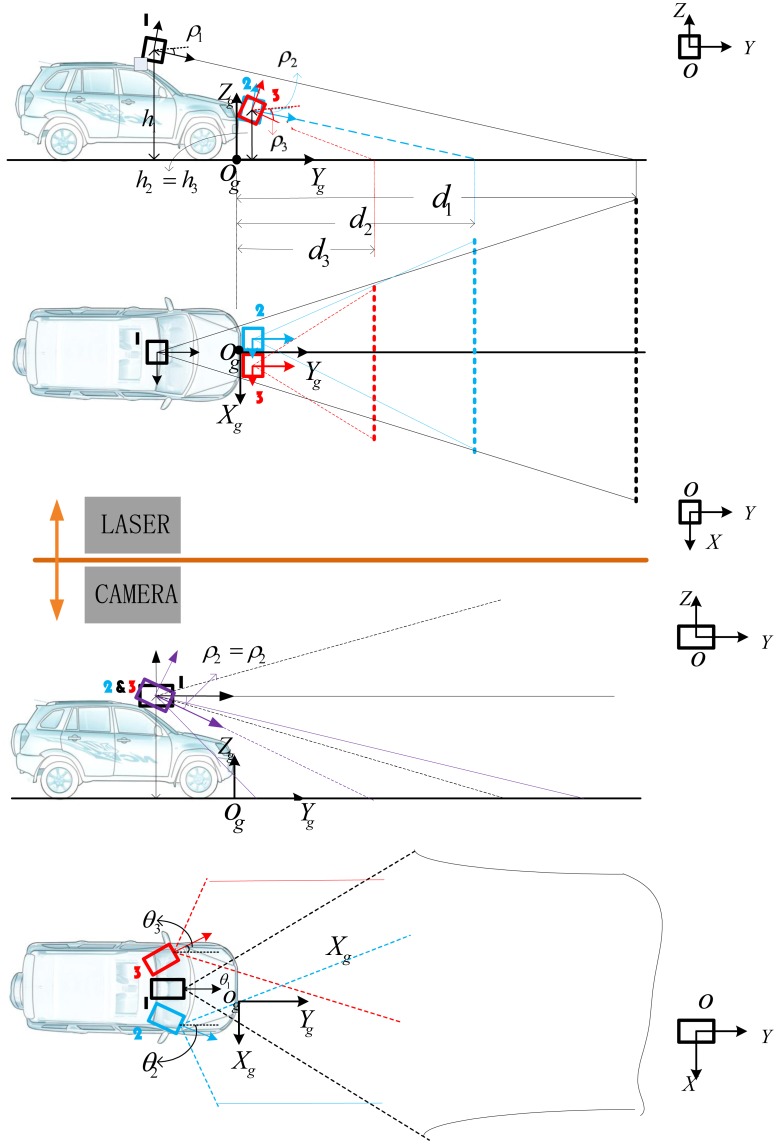
Lasers and cameras layout.

The layout of the sensors for IVS should enable a wide view including not only the front view but also the left and right sides of the vehicle. Compared with the two successful vehicle in DAPRA Challenge, *i.e.*, BOSS [43] from CMU and Stanley [44] from Stanford University, our system uses lower cost sensors instead of the high-end laser scanners such as Velodyne and fixes several sensors in the front part of the vehicle to cover the area close to the vehicle. Our detection system arranges the layout of lasers and cameras in such a way that guarantees our range of perception should cover not only the front view of the ego vehicle but also the left and right view. This arrangement can deal with the situation where the vehicle prepares to drive through a turn with high speed. Figure 2 shows the positions and coverage areas of the sensors. Three laser scanners are marked by 1, 2 and 3 in the upper figure. Laser 1 is mounted on the roof and Laser 2 and Laser 3 are mounted on the head of the vehicle, tilted downward to scan the road ahead. We can adjust the pitch angles *ρ*_1_, *ρ*_2_ and *ρ*_3_ in order that the lasers can touch different distances ahead our vehicle. Three cameras with different pitch angles and heading angles are used for curb finding. When vehicle is traveling roughly along the straight line, the middle camera is used for lane detection. When it comes to turning, two aside cameras are chosen in order to cover the closer area around the vehicle. Data from different sensors will be transformed to the unique vehicle coordinate. Calibration is performed using OPENCV functions [45] and the Camera Calibration Toolbox for MATLAB. The algorithm used is taken mainly from [46].

## 3. Road Curb Detection

### 3.1. Z-Variance Based Road Curb Detection

The laser scanner used for road shoulder detection is slanted down. The proposed method assumes that the road surface is flat. With this hypothesis, the elevation variance of the points on road surface is low, while the variance of Z value is high on the road boundary or curb. All the laser points are translated to the vehicle coordinate. Median filter is applied to filter out some tiny objects on the road such as leaves and road crack. The Z-variance of the *i*th point will be calculated by

(1) $$D_{i}=\frac{1}{9}\sum_{i-4}^{i+4}Z_{k}$$

The algorithm step is as follows:

1. Calculate the Z-variance of all points.
2. Select the points with Z-variances above the threshold *t*, and the segment between these two points with length wider than the vehicle will be selected as candidate road section.
3. Compare the mean value of height *H*, distance *D* between head of vehicle and midpoint of one section, then calculate weights for all candidate road sections by the following equation:
  (2) $$\begin{array}{cc} W_{i}=\alpha \cdot & e^{-\left|\frac{H-H_{\text{min}}}{H_{\text{min}}}\right|} \end{array}+(1-\alpha )e^{-\left|\frac{D-D_{\text{min}}}{D_{\text{min}}}\right|}$$
  where *H_min_* is the minimum height and *D_min_* is the minimum distance, and *α* is a weighting factor. *W_i_* ranges from 0 to 1.
4. The candidate road section with highest weight is considered as the real road which is expressed as pointpair, that is, left point (*X_l_,Y_l_*) and right point (*X_R_, Y_R_*).

### 3.2. Multi-Laser Based Road Curb Fitting

**Figure 3.**
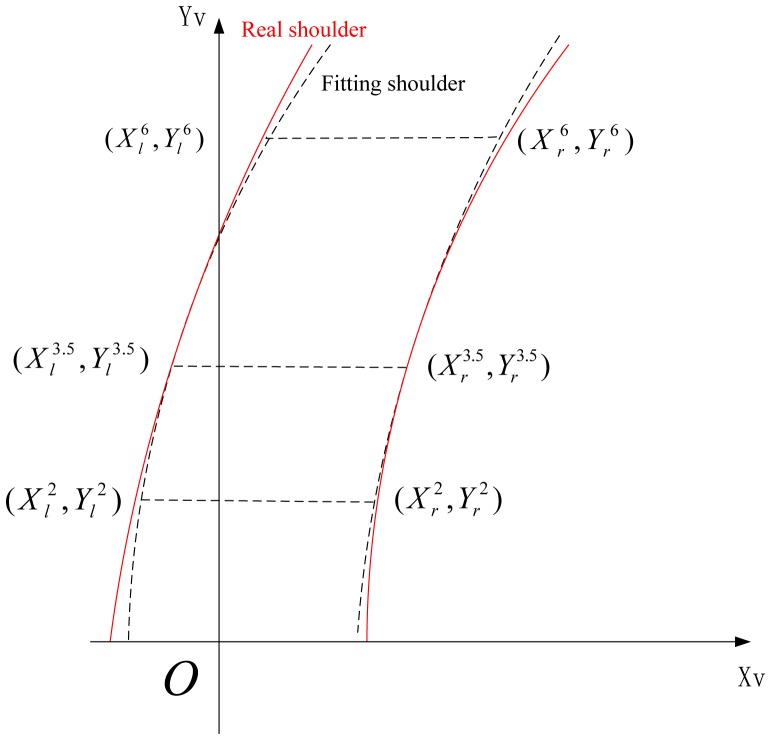
Road curb fitting.

To obtain the road boundary, only one single scan laser is not enough. Multiple lasers are combined to settle this problem. Three SICK laser scanners are used with scan range 2 m, 3.5 m and 6 m respectively. Road curb detection described above will be carried out with each laser dependently. Consequently, we can get three point-pairs which can be divided into left points ((*X_L_*^2^,*Y_L_*^2^),(*X_L_*^3.5^,*Y_L_*^3.5^) and (*X_L_*^6^,*Y_L_*^6^)) and right points ((*X_R_*^2^, *Y_R_*^2^),(*X_R_*^3.5^, *Y_R_*^3.5^) and (*X_R_*^6^, *Y_R_*^6^)). Finally, a parabola is used to fit the points on the same side, see Figure 3.

(3) $$x=\alpha +by+cy^{2}$$

## 4. Lane Detection

**Figure 4.**
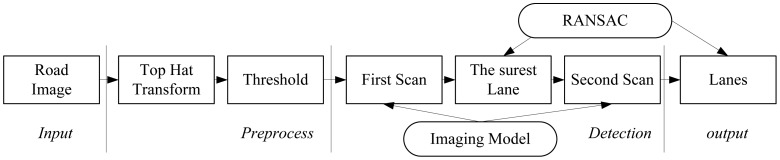
Flowchart of two scans based lane detection method.

For structured roads, this paper proposes a two scans method to detect multiple lanes. Figure 4 is the proposed flow chart of multiple lanes detection method. Road image from top-middle camera is first preprocessed by top-hat transform and threshold. In mathematical morphology, top-hat transform is an operation that extracts small elements and details from given images. The top-hat extracts the objects that have not been eliminated by the opening. That is, it removes objects larger than the structuring element.

### 4.1. Imaging Model

**Figure 5.**
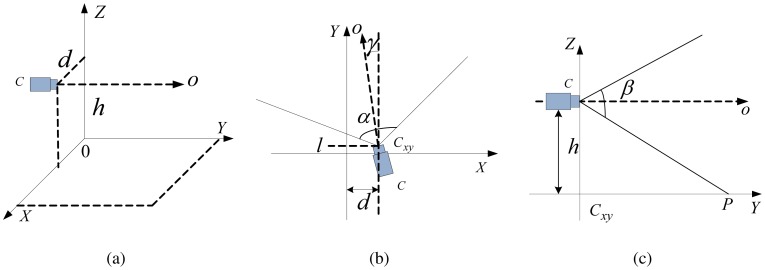
Imaging Model. (**a**) The *W* space. (**b**) The *xy* plane in the *W* space. (**c**) The *yz* plane in the *W* space.

Using the image model, we can rebuild the model of the lane plane in the 3D world space from the image in the 2D image space based on the inverse perspective mapping (IPM), and finally obtain the real width of lane markings and distance between two adjacent lane markings. The proposed image model is shown in Figure 5. *W* = (*X, Y, Z*) ∈ *E*^3^ denotes the world coordinate system *WCS* and *I* = (*u, v*) ∈ *E*^2^ denotes the image coordinate. Camera is located in *C*(*d*, 0, *h*) ∈ *W*, *h* is the height of the camera from the ground. Optical axis is parallel to the ground, *γ* is the angle between optical axis and the lane. *α* is horizontal view angle of the camera and *β* is vertical view angle. The mapping from *W* to *I* is given in Equation (4) and the mapping from *I* to *W* is given in Equation (5), where *H_I_* and *W_I_* respectively represents horizontal resolution and vertical resolution of the camera, which can be acquired by calibration. The width of the lane marking decreases with increasing distance to the camera in perspective view. Based on imaging model, we can get the real distance Δ*X* in the *WCS* coordinate when the distance is Δ*u* in the line *v* in the image coordinate. The relationship is given in Equation (7).

(4) $$\begin{array}{r} v=\frac{H_{I}}{2}(1-\frac{h}{Y\times \tan\frac{\beta }{2}\times \cos\gamma })\\ u=\frac{W_{I}}{2}(1-\frac{X}{Y\tan\frac{\alpha }{2}}) \end{array}$$

(5) $$\begin{array}{r} X=\frac{Y\tan\frac{\alpha }{2}}{\text{cot}\gamma }(1-\frac{2u}{W_{I}})\\ Y=\frac{h\times H_{I}}{(H_{I}-2v)\times \tan\frac{\beta }{2}\times \cos\gamma } \end{array}$$

(6) $$\Delta u=\frac{\Delta X\times W_{I}(2v-H_{I})\text{cot}\gamma \text{cos}\gamma \text{tan}\frac{\beta }{2}}{2\times hH_{I}\times \text{tan}\frac{\alpha }{2}}$$

### 4.2. Two Scans Based Method for Multi-Lane Detection

**Figure 6.**
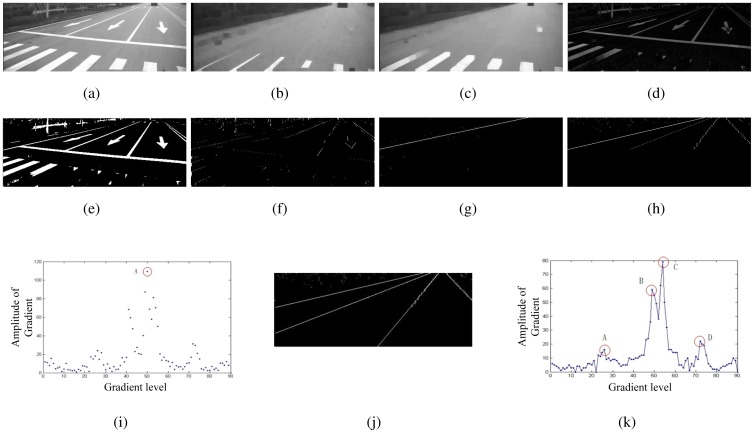
Step by step results by proposed lane detection. (**a**) Original Image. (**b**) Image after open operation. (**c**) Image after dilate operation. (**d**) Image after top-hat transform. (**e**) Threshold. (**f**) First scan. (**g**) RANSAC. (**h**) Second scan. (**i**) Gradient contribution function after first scan. (**j**) RANSAC. (**k**) Gradient contribution function after second scan.

After preprocessing, the gradient of each pixel will be calculated as follows:

(7) $$\nabla I(x,y)=(\frac{\partial I}{\partial x},\frac{\partial I^{T}}{\partial y})\approx {\left(D_{x},D_{y}\right)}^{T}$$

where *D_x_* and *D_y_* denote the gradient in *x* direction and *y* direction respectively. First, we want to get a most obvious lane, called the surest lane, based on the edge distribution function(EDF). EDF is the histogram of the gradient magnitude with respect to the orientation. We can estimate the magnitude and orientation by Equation (8). To compute this histogram, the angle *θ*(*x, y*) with the range [−90°, 90°] were quantized in 90 subintervals at a step of 2°. The surest lane is defined as the maximum value of the histogram. Figure 6(g) shows the RANSAC line fitting of the surest lane after the first scan. Starting from the surest lane, we can do the second scan. Other lanes could be fitted with the same method. Figure 6(j) shows the results of multiple lane detection. Figure 6(i,k) presents the global maxima and local maxima.

(8) $$\begin{array}{r} \left|\nabla I(x,y)\right|=\left|D_{x}\right|+\left|D_{y}\right|\\ \theta (x,y)=\tan^{-1}\frac{D_{y}}{D_{x}} \end{array}$$

## 5. Traffic Sign Detection and Classification

**Figure 7.**
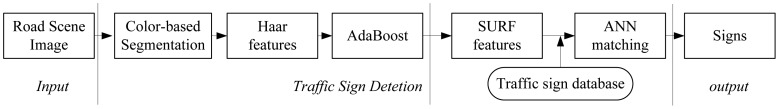
Flow chart of traffic signs recognition system.

**Figure 8.**
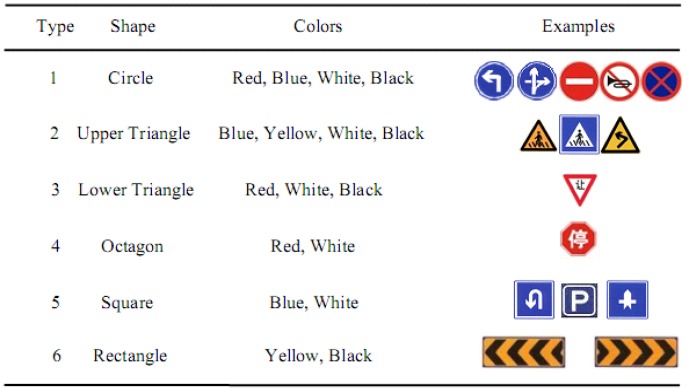
Traffic Signs Classes.

The proposed sign detection and recognition method includes two parts. The detection part is based on color segmentation, Haar-like wavelet features and AdaBoost classifier. The recognition part is based on feature matching method with the Speeded Up Robust Features (SURF). Figure 7 is the flow chart of the traffic sign recognition system. Because Haar-like features are features of gray images, the detection method we proposed here is mainly based on the gray information. Since the shape information can mainly affect the Haar-like features, the main traffic signs that this paper copes with can be divided into six classes based on the shape, as shown in Figure 8.

### 5.1. Color-Based Segmentation

The color-based segmentation includes two steps: (1) color quantization, (2) ROI locking. In the first step, we extract the target color pixels. In the next step, we get the ROI from the pixels based on constraints on bounding box of the connected-components of the pixels. The main color includes: red, blue, yellow, white and black. In our detection method, we focus on the three colors: red, blue and yellow. The RGB color model is highly related to the light intensity. HSV color model is applied in this paper.

**Figure 9.**
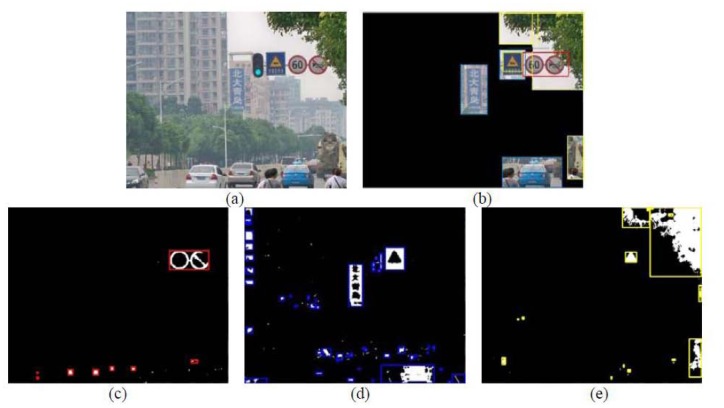
Color quantization and ROI locking. (**a**) original image, (**b**) ROI locking, (**c**) red segmentation, (**d**) blue segmentation, (**e**) yellow segmentation.

According to Table 1, we can get the red, blue and yellow pixels from the original image. After the color segmentation, the detected pixels can form some connected regions, then we can get the enclosing rectangles (ER) of them. Based on some constraints on ER, we can wipe off many noise regions. First, the ER smaller than 20 × 20 pixels are considered as noise and not processed further. Second, the aspect ratio of ER is limited to 2. Third, the saturation of ER is no less than 0.5. The rest of ERs will be ignored. Figure 9 shows the results of three color segmentation and ROI locking.

### 5.2. AdaBoost for Traffic Sign Detection

The AdaBoost algorithm is a classifier learning method which combines a set of weak classifiers to construct a strong classifier and then assembles some strong classifiers to a cascade classifier. Feature selection is crucial for classifier. Motivated by the work of Tieu and Viola [47], we use extended Haar-like features to train AdaBoost classifier for traffic signs detection.

(9) $${\text{feature}}_{i}=\sum_{i\in (i,n)}\omega_{i}\times \text{RectSum}(r_{i})$$

where *ω_i_* denotes the weight of rectangle, *RectSum*(*r_i_*) is the integral of image by surrounded by rectangle *r_i_*, *feature_j_* is the *j_th_* feature, *n* is arbitrarily chosen that represents the number of rectangles consisting of *feature_j_*.

### 5.3. SURF Matching for Classification

The proposed recognition method includes three steps: image scaling, SURF features extraction, features matching. The detected targets found in detection stage will be normalized to be of the same size (100 × 100) as the template which will be matched. Though SURF is a scale invariant feature, in this step we will make sure that the true sign contains enough features to be matched with the template sign. If the number of matched points is lower than a certain value, the candidate will be discarded as a noise. In order to make sure the certain value is adequate for all candidates, the image scaling is necessary. In this paper, we use bilinear interpolation for image scaling. Once the image is normalized, the SURF descriptor can be used for exacting the scale and rotation invariant features.

SURF [48,49] detector is chosen instead of the often used SIFT detector. SURF is developed to run substantially faster but possess comparable performance than SIFT. The resulting descriptor vector for all 4 × 4 sub-regions is of length 64. More details about SURF can be found in [48] and [49].

**Figure 10.**
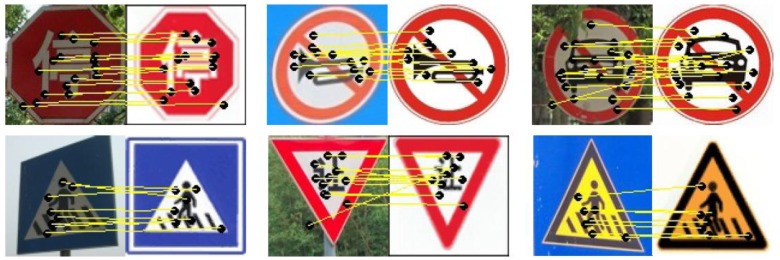
SURF feature matching. The number of match points is 16, 11, 24, 7, 12, 7 according to priority.

Because we have many template signs to be matched, in order to reduce the matching time, all the template signs are divided into six groups based on the color and the trained Adaboost classifiers. We used Approximate Nearest Neighbor (ANN) [50] algorithm for matching. SURF features are first extracted from all the template signs which will be divided into eight groups and stored in a database. Then a candidate image is matched by individually comparing each feature of the candidate with the special database; the selection is made based on the classifier used and color information and the features are matched based on ANN. The image in the template database that gives the maximum number of matches with the candidate image is the target class. Figure 10 shows some match results between the candidate signs and template signs. See [51] for more details about the algorithm.

## 6. Results and Discussion

### 6.1. Road Curb Detection

**Figure 11.**
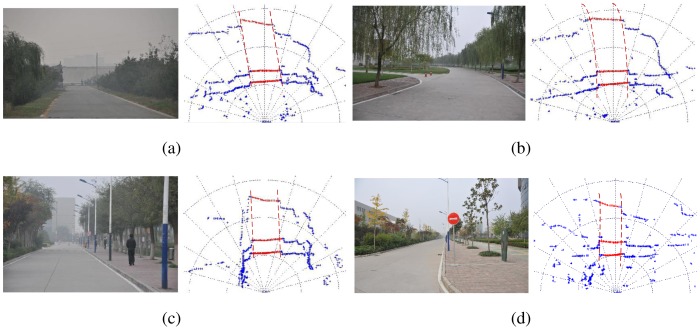
Some results of the road curb detection.

In order to test the curb detection algorithm, we collected the synchronous laser data and the image data of the whole route in the Future Challenge 2010. The data set contains 9,230 frames as a combination of three laser scanners. If the road curb detected from the laser data is close to the scene in image, we consider it as true position. The final accuracy can reach 82%. Figure 11 shows some results of the proposed road curb detection. The point in red denotes the road segment point obtained from our curb detection method. The red dashed line represents the fitting boundary based on the curb points.

### 6.2. Lane Detection

The algorithm takes the mobile laboratory SmartV-II (Figure 1(b)) Wuhan University as the platform. The test image data is acquired by the analog Video Camera, which is mounted on the top of the Chery SUV with a fixed strut. The size of the recorded images is 640 × 480. For some special reason, we transform the video to 388 × 332. We tested the system under a variety of different road conditions, including structured road and unstructured road. The test data contains 15 videos and 4,319 frames in total, among which unstructured road (without lanes) consisting 2,891 frames and unstructured road (without lanes) consisting 1,428 frames. All the videos are taken on urban roads in Wuhan and Xi'an City, China. The average error rate under different conditions is lower than 9%. The average processing time is 20 ms per frame on a Pentium E5200 2.5 GHz computer. For comparison, we implemented the Canny/Hough Estimation of Vanishing Points (CHEVP) algorithm [13]. Wang *et al.* proposes the CHEVP algorithm to initialize their B-Spline SNAKE tracking algorithm. Here, we just compare the detection algorithm instead of tracking. For all the 4,319 frames, the correct detection of CHEVP is lower than 30%, and for the 1,428 structured road frames, the correct detection is no more than 50%. The main reason is the Hough failed to grab many unobvious lines.

**Figure 12.**
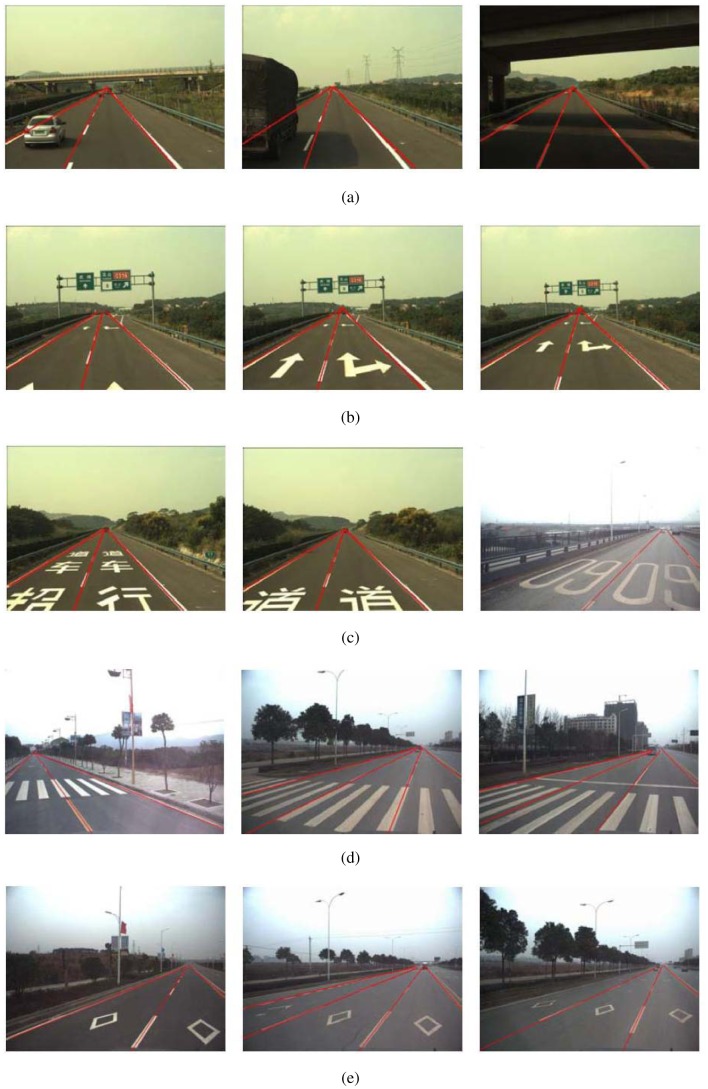
Some examples of lane detection results.

Figure 12 shows some results from the front camera under different road conditions. Figure 12(a) shows the roads with vehicle or shadow. Figure 12(b) shows the highway with orientation arrows markings. Figure 12(c) shows the highway with crosswalk warning markings. Figure 12(d) is the road with crosswalk markings. Figure 12(e) shows the road with pavement lettering markings.

### 6.3. Traffic Sign Detection and Recognition

The test image data is acquired by the CCD Video Camera which is mounted on the top of the Chery SUV with a fixed strut. The size of the recorded images is of 640 × 480. We tested the system under a variety of different conditions. To evaluate the performance of the proposed method, 200 images were taken as test images, in which there are 281 traffic signs.

**Figure 13.**
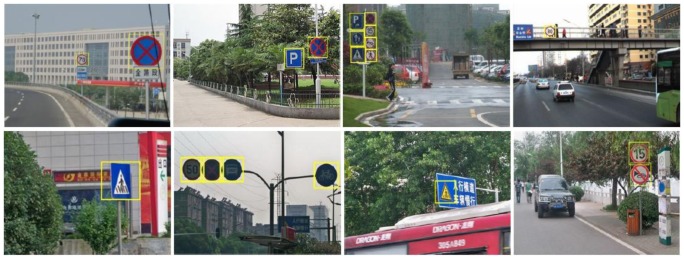
Detection of traffic signs under various conditions.

In this paper, six classifiers were trained for the six classes of signs listed in Figure 8. For all the classifiers, the number of position samples (PS) and negative samples (NS) are listed in Table 2. Our method can detect road signs in 50 ms. In the 281 signs, there are 265 signs being correctly detected, 14 signs being missed, and 2 signs being false alarm. Thus the detection rate is 94.3%, demonstrating that the proposed detection method is effective and efficient. Some detection results are shown in Figure 13 to demonstrate that our method is insensitive to many complex conditions.

The 265 detected traffic signs are used to evaluate the performance of the proposed method. Among the 265 signs, 244 signs are correctly classified and 14 signs are falsely classified. The recognition accuracy is 92.7%.

## 7. Conclusions

In this paper, we propose a real-time traveling environment perception system for autonomous vehicle navigation. Our system makes use of the good aspects of laser and camera respectively. At the same time, the combination of multiple lasers and multiple cameras can cover all the front view of ego vehicle, and their information fusion can deal with tough situations. The functions of our perception system include road shoulder detection, lane detection and traffic sign recognition. Many experiment results show that our system is reliable in synthetic urban environment. Our future work will also introduce the Velodyne laser scanner to deal with more complex road conditions and make use of SLAM to develop our IVS.

## Figures and Tables

**Table 1.** Color quantization.

	Red	Blue	Yellow
Saturation	*S* > 0.2	*S* > 0.2	*S* > 0.2
Hue	0 < *H* < 10320 < *H* < 360	200 < *H* < 270	20 < *H* < 100

**Table 2.** The number of PS and NS for the six trained classifiers.

	C1	C2	C3	C4	C5	C6
PS	3,125	1,276	794	648	963	346
NS	5,200	2,300	16,00	1,600	1,600	1,000
